# Novel Genes Potentially Involved in Fibroblasts of Diabetic Wound

**DOI:** 10.1155/2021/7619610

**Published:** 2021-12-07

**Authors:** Weirong Zhu, Qin Fang, Zhao Liu, Qiming Chen

**Affiliations:** ^1^Department of Hand and Foot Surgery, Huizhou Municipal Central Hospital, Huizhou 516001, China; ^2^Department of Breast Surgery, Huizhou Municipal Central Hospital, Huizhou 516001, China

## Abstract

Fibroblasts are the essential cell type of skin, highly involved in the wound regeneration process. In this study, we sought to screen out the novel genes which act important roles in diabetic fibroblasts through bioinformatic methods. A total of 811 and 490 differentially expressed genes (DEGs) between diabetic and normal fibroblasts were screened out in GSE49566 and GSE78891, respectively. Furthermore, the Kyoto Encyclopedia of Genes and Genomes (KEGG) pathways involved in type 2 diabetes were retrieved from miRWalk. Consequently, the integrated bioinformatic analyses revealed the shared KEGG pathways between DEG-identified and diabetes-related pathways were functionally enriched in the MAPK signaling pathway, and the MAPKAPK3, HSPA2, TGFBR1, and p53 signaling pathways were involved. Finally, ETV4 and NPE2 were identified as the targeted transcript factors of MAPKAPK3, HSPA2, and TGFBR1. Our findings may throw novel sight in elucidating the molecular mechanisms of fibroblast pathologies in patients with diabetic wounds and targeting new factors to advance diabetic wound treatment in clinic.

## 1. Introduction

As the global population ages, the incidence of diabetes is rapidly increasing during recent decades [1]. Diabetic foot ulcers (DFUs) are one of the most common and serious complications of diabetes. It was reported that the incidence of DFUs was up to 4% in diabetes [2]. The mechanism of DFUs remains unclear, and many factors contributed to the delayed healing of it, throwing a significant burden on patients with diabetic wound [3]. Early diagnosis and intervention of diabetic wound are important for reversing the poor prognosis of DFUs [4]. Unfortunately, few distinctive diagnostic biomarkers have been reported and demonstrated in diabetic wound. Thus, it is of great necessity to screen out the novel diagnostic biomarkers involved in the development of diabetic wound.

Fibroblasts are the essential cell type of skin, highly involved in the wound regeneration process, and acted in wound healing by interacting with other cells including keratinocytes and endothelial cells [5]. Exosomal miR-20b-5p derived from the high-glucose impaired fibroblast proliferation and differentiation, and delayed diabetic wound healing, suggesting the crucial role of fibroblasts in diabetic wound healing [6]. Furthermore, accumulative evidences have demonstrated the important role of genetic and epigenetic regulation in diabetic wound healing [7, 8].

In this study, we sought to identify the DEG modulation in diabetic fibroblasts by using bioinformatic methods. These findings may provide useful insights into understanding the molecular mechanisms of fibroblast pathologies in patients with DFUs.

## 2. Materials and Methods

### 2.1. DEG Identification

Microarray data of datasets comparing diabetes and the healthy controls were screened out from the Gene Expression Omnibus database (GEO, http://www.ncbi.nlm.nih.gov/geo). DEGs were performed by Limma in R, and *p* values < 0.05 were considered as statistically significant. R package pheatmap was used to visualize Log2 mRNA gene expression. Using Circlize and ComplexHeatmap in R, common DEGs from different datasets were identified and visualized. The circular visualization of chromosomal information of common DEGs was achieved with circular visualization in R.

### 2.2. GO and KEGG Analyses

DAVID, an online bioinformatics tool, was used to perform GO and KEGG analyses. The top ten GO terms in biological process, molecular function, and cellular component and top five KEGG pathways were identified using the enrichment analysis. The result of enrichment analysis of hub genes was visualized with GOplot. DEGs were imported into Search Tool for the Retrieval of Interacting Genes (STRING) to construct the PPI network. Then, the TSV file of PPI network was imported into Cytoscape 3.7.2. The interactions between enriched KEGG pathways were calculated and visualized by Cytoscape 3.7.2.

### 2.3. Retrieval of KEGG Pathways Involved in Type 2 Diabetes and Calculation of Shared Pathways between Enriched Pathways and Type 2 Diabetes

miRWalk is an online bioinformatics atlas tool. In this study, the KEGG pathways involved in type 2 diabetes were retrieved from miRWalk. Then, the intersection of enriched KEGG pathways (*p* ≤ 0.05) and type 2 diabetes-related KEGG pathways was obtained with Draw Venn Diagram (http://bioinformatics.psb.ugent.be/webtools/Venn/). The top shared KEGG pathway with the smallest *p* value was selected. The enriched DEG-related part of the KEGG pathway was established with the PPT drawing tool.

### 2.4. Targeted Transcript Factor Prediction

http://amp.pharm.mssm.edu/Enrichr/, the online predicting tool, was used to predict targeted transcript factors of enriched DEGs in the shared KEGG pathway. The prediction result was visualized by Gephi.

## 3. Results

### 3.1. DEG Identification

**Figure 1:**
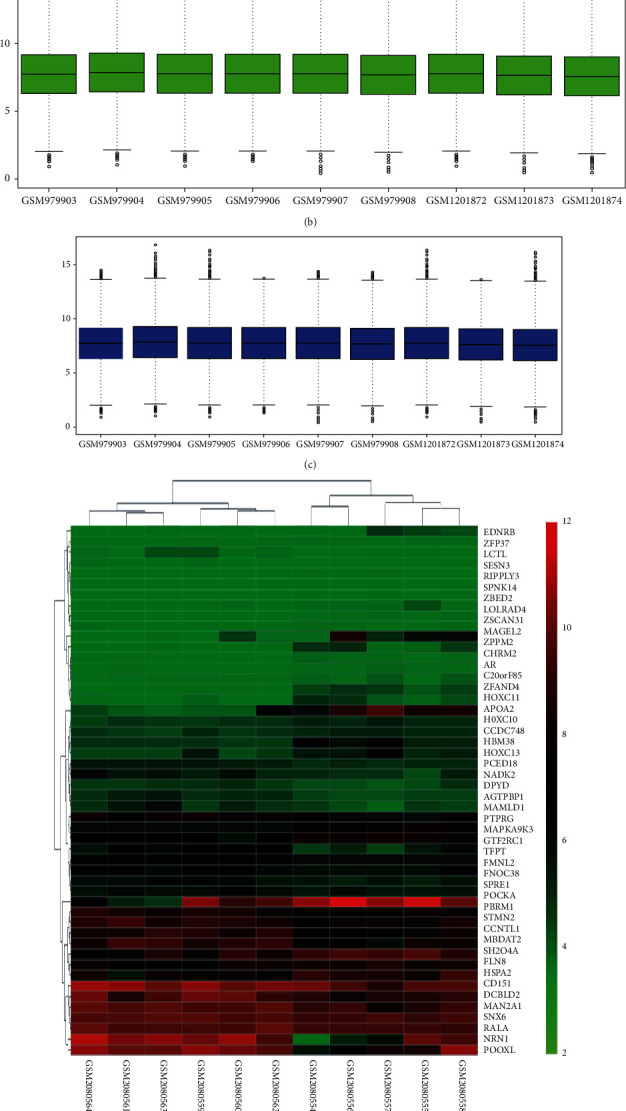
Heatmap of the DEGs between type 2 diabetes and normal people in GSE49566 and GSE78891: (a) heatmap clustering of the DEGs in GSE49566; (b) gene expression information of each sample before standardization in GSE49566; (c) gene expression information of each sample after standardization in GSE49566; (d) heatmap clustering of the DEGs in GSE78891; (e) gene expression information of each sample before standardization in GSE78891; (f) gene expression information of each sample after standardization in GSE78891.

**Figure 2:**
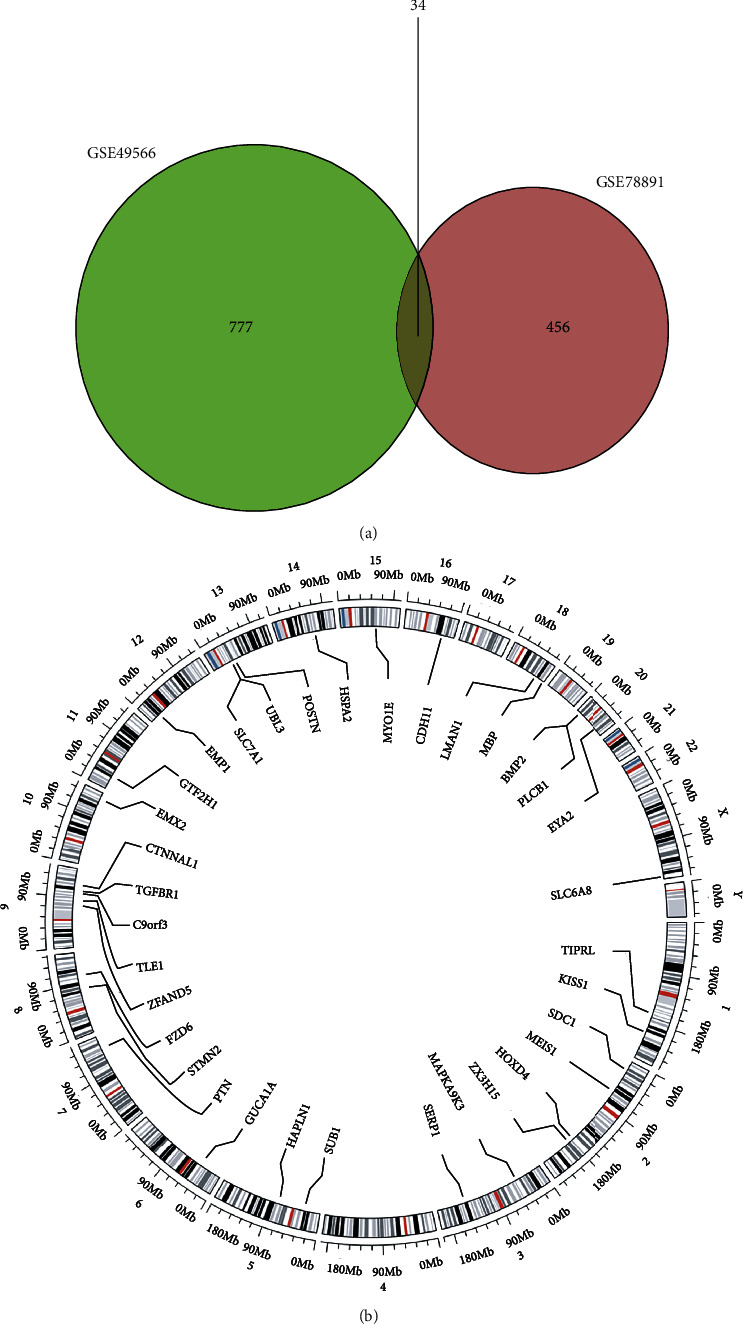
The information of common DEGs: (a) 34 common DEGs were identified between the two datasets; (b) the gene position information of the 34 common DEGs.

Datasets of GSE49566 and GSE78891 were obtained from GEO, which are the genes from human skin fibroblasts (Figure 1). There were three type 2 diabetes samples and six normal in GSE49566. There were six type 2 diabetes samples and five normal in GSE78891. 446 upregulated and 365 downregulated DEGs were identified in GSE49566. 242 upregulated and 248 downregulated DEGs were identified in GSE78891. Totally, there were 34 common DEGs identified. They were STMN2, HAPLN1, PTN, POSTN, MAPKAPK3, CDH11, TLE1, ZFAND5, C9orf3, EMX2, TIPRL, MEIS1, FZD6, SLC6A8, SLC7A1, TGFBR1, EMP1, HSPA2, PLCB1, KISS1, HOXD4, EYA2, SERP1, UBL3, GTF2H1, MYO1E, LMAN1, BMP2, CTNNAL1, SDC1, GUCA1A, SUB1, ZC3H15, and MBP (Figure 2).

### 3.2. GO and KEGG Pathway Enrichment Analysis

**Figure 3:**
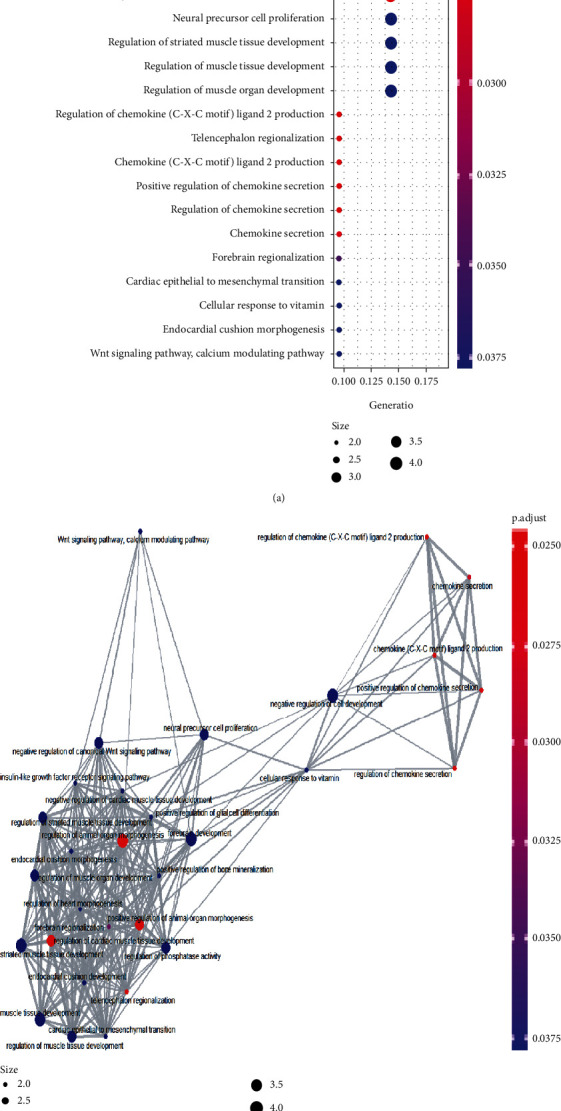
GO and KEGG enrichment analysis results: (a) count number, gene ratio, and adjusted *p* value of common DEGs; (b) the interaction relationship of the GO and KEGG terms.

GO analysis results showed that common DEGs were significantly enriched in skeletal system development, cell surface, protein binding, mesenchymal differentiation, pathway-restricted SMAD protein phosphorylation, cardiac epithelial to mesenchymal transition, heart development, in utero embryonic development, regulation of transcription, DNA-templated, and skeletal system morphogenesis (Table 1). KEGG pathway analysis showed that the common DEGs were significantly enriched in a pathway in cancer, signaling pathways regulating pluripotency of stem cells, Hippo signaling pathway, MAPK signaling pathways, and basal cell carcinoma pathway (Table 2). The information and interaction of the GO and KEGG terms are demonstrated in Figure 3.

### 3.3. Retrieval of KEGG Pathways Involved in Type 2 Diabetes and Calculation of Shared Pathways between Enriched Pathways and Type 2 Diabetes

**Figure 4:**
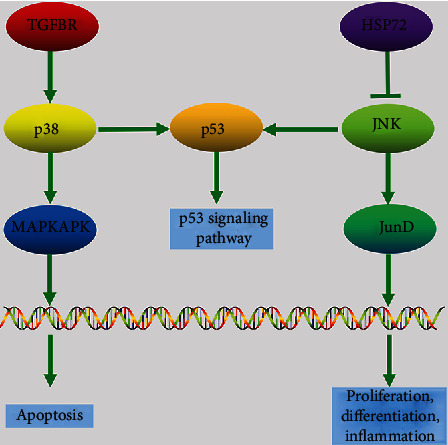
The part of the MAKP signaling pathway related to the common DEGs. MAPKAPK3, HSPA2, TGFBR1, and p53 signaling pathways were involved, resulting in apoptosis, proliferation, differentiation, and inflammation.

The KEGG pathways linked with type 2 diabetes were obtained from miRWalk. They are listed in Table 3. Totally, there were 44 KEGG pathways involved in the development of type 2 diabetes. The common KEGG pathway between DEGs and type 2 diabetes with the highest *p* value was the MAPK signaling pathway. The part of the MAPK signaling pathway related to the DEGs was established (Figure 4). MAPKAPK3, HSPA2, TGFBR1, and p53 signaling pathways were involved.

### 3.4. Targeted Transcript Factor Prediction

**Figure 5:**
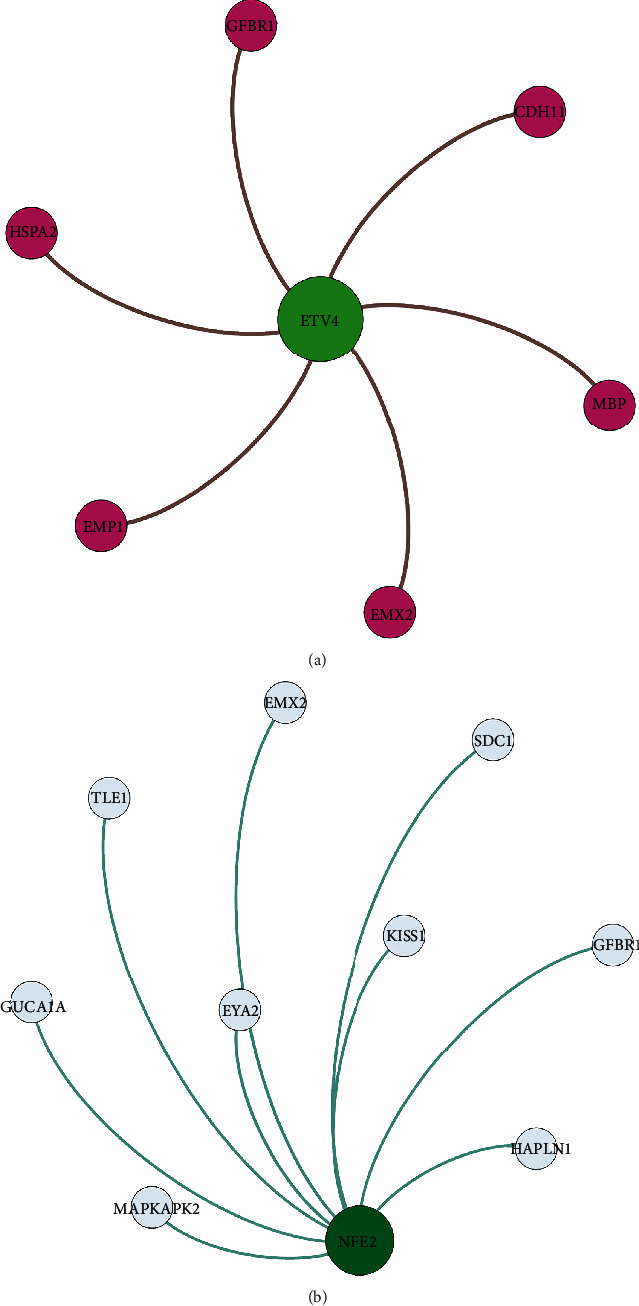
Targeted transcript factor prediction of the DEGs in the MAKP signaling pathway. They were ETV4 and NPE2. ETV4 may target TGFBR1, HSPA2, EMP1, EMX2, MBP, and CDH11. NPE2 may target MAPKAPK3, GUCA1A, TLE1, EYA2, EMX2, SDC1, KISS1, TGFBR1, and HAPLN1.

The targeted transcript factors of MAPKAPK3, HSPA2, and TGFBR1 were obtained from http://amp.pharm.mssm.edu/Enrichr/, which indicated ETV4 and NPE2 were the potential ones. The relationship of transcript factors, DEGs, and other targeting genes is shown in Figure 5.

## 4. Discussion

High risk of wound infection and healing failure was found in diabetes, and the abnormal function of fibroblasts was assumed as a major issue contributing to the delayed wound healing [9–11]. Noticeably, fibroblasts exert an important role in wound inflammatory response by release of various antibacterial regulators, providing a robust defense of skin against infections [12–14]. Diabetes patients are susceptible to infections due to the dysregulated function of the T cells, leading to the overactivated tissue inflammation. In this bioinformatic research, functional enrichment analysis was performed, and the systematic results suggested that the highest *p* value was the MAPK signaling pathway among DEGs in fibroblasts. And the regulatory roles for diabetic wound healing were identified in MAPKAPK3, HSPA2, and TGFBR1.

Phosphorylation of transcription is one of the modifications of MAPK-dependent regulation in cellular responses [15]. Three subfamilies were found in the MAPK signaling pathway, including the extracellular-signal-regulated kinases (ERK MAPK, Ras/Raf1/MEK/ERK), the c-Jun N-terminal or stress-activated protein kinases (JNK, SAPK), and p38 [16–18]. Once the pathway was activated, a number of downstream target kinases including MAPKAPK3 could be activated [19]. Recently, some researchers have fabricated an in situ injectable hydrogel which can markedly accelerate diabetic wound healing through activating the TGF-*β*/MEK/MAPK signaling pathway [20]. Similarly, Qian et al. demonstrated that protein tyrosine phosphatase 1B was capable to enhance fibroblast proliferation and mitigation via activation of the MAPK/ERK pathway, thereby promoting diabetic wound healing [21]. In the current study, we found a consistent result that the MAPK signaling pathway plays a key role in the regulation of diabetic wound healing, and MAPKAPK3, HSPA2, and TGFBR1 are the potentially critical genes in this regulation process. Moreover, to uncover the potential targeted transcript factors of MAPKAPK3, HSPA2, and TGFBR1 genes, we used the online software (Enrichr, http://amp.pharm.mssm.edu/Enrichr/) and the results suggested that ETV4 and NPE2 were the potential transcript factors for these genes. Thus, it was assumed that ETV4 and NPE2 may exert a critical role in the regulation of diabetic wound healing.

Some limitations also existed in this bioinformatic research. First, the current results were based on a public database and only two datasets were included in our study; the sample size should be enlarged to minimize the possible confounding factors. Furthermore, this is a pure bioinformatic research; more experimental validation is needed to confirm the candidate pathways and their potential transcript factors. Moreover, clinical specimens of different degrees of DFUs should be collected to validate our current findings.

## 5. Conclusions

Our findings suggested a functionally enriched MAPK signaling pathway, with a focus on the potential role of ETV4 and NPE2 in the regulation of diabetic wound regeneration. The current study may provide novel therapeutic targets in diabetic wound treatment.

## Figures and Tables

**Table 1:** Functional enrichment analysis of the DEGs. Top 10 terms were selected according to *p* value.

Term	Name	Count	*p* value	Genes
GO:0001501, BP	Skeletal system development	5	2.3*E* − 5	POSTN, BMP2, CDH11, TGFBR1, HAPLN1
GO:0009986, CC	Cell surface	5	3.7*E* − 3	BMP2, FZD6, PTN, HSPA2, TGFBR1
GO:0005515, MF	Protein binding	18	6.1*E* − 3	EMX2, POSTN, TLE1, EYA2, FZD6, ZFAND5, STMN2, HSPA2, SLC7A1, TGFBR1, KISS1, LMAN1, BMP2, MEIS1, MAPKAPK3, TIPRL, MBP, PLCB1
GO:0048762, BP	Mesenchymal cell differentiation	2	9.9*E* − 3	BMP2, TGFBR1
GO:0060389, BP	Pathway-restricted SMAD protein phosphorylation	2	1.6*E* − 2	BMP2, TGFBR1
GO:0060317, BP	Cardiac epithelial to mesenchymal transition	2	1.6*E* − 2	BMP2, TGFBR1
GO:0007507, BP	Heart development	3	2.2*E* − 2	BMP2, PTN, TGFBR1
GO:0001701, BP	In utero embryonic development	3	2.3*E* − 2	BMP2, ZFAND5, TGFBR1
GO:0006355, BP	Regulation of transcription, DNA-templated	6	3.5*E* − 2	EMX2, MEIS1, BMP2, TLE1, EYA2, TGFBR1
GO:0048705, BP	Skeletal system morphogenesis	2	3.9*E* − 2	ZFAND5, TGFBR1

BP: biological process; MF: molecular function; CC: cellular component.

**Table 2:** Pathway enrichment analysis of the DEGs. Top 5 KEGG pathways were selected according to *p* value.

Term	Name	Count	*p* value	Genes
hsa05200	Pathways in cancer	4	8.4*E* − 3	BMP2, FZD6, PLCB1, TGFBR1
hsa04550	Signaling pathways regulating pluripotency of stem cells	3	1.1*E* − 2	MEIS1, BMP2, FZD6
hsa04390	Hippo signaling pathway	3	1.2*E* − 2	BMP2, FZD6, TGFBR1
hsa04010	MAPK signaling pathway	3	3.3*E* − 2	MAPKAPK3, HSPA2, TGFBR1
hsa05217	Basal cell carcinoma	2	6.1*E* − 2	BMP2, FZD6

KEGG: Kyoto Encyclopedia of Genes and Genomes.

**Table 3:** Information on KEGG pathways linked with diabetes type 2.

Code	KEGG
hsa00061	Fatty acid biosynthesis
hsa04910	Insulin signaling pathway
hsa01100	Metabolic pathways
hsa00640	Propanoate metabolism
hsa00620	Pyruvate metabolism
hsa04920	Adipocytokine signaling pathway
hsa03320	PPAR signaling pathway
hsa04930	Type II diabetes mellitus
hsa05332	Graft versus host disease
hsa04672	Intestinal immune network for IgA production
hsa05322	Systemic lupus erythematosus
hsa04660	T cell receptor signaling pathway
hsa04940	Type I diabetes mellitus
hsa05416	Viral myocarditis
hsa05330	Allograft rejection
hsa05320	Autoimmune thyroid disease
hsa04514	Cell adhesion molecules (CAMs)
hsa04920	Adipocytokine signaling pathway
hsa04512	ECM receptor interaction
hsa04640	Hematopoietic cell lineage
hsa03320	PPAR signaling pathway
hsa05320	Autoimmune thyroid disease
hsa04514	Cell adhesion molecules (CAMs)
hsa04660	T cell receptor signaling pathway
hsa04010	MAPK signaling pathway
hsa01100	Metabolic pathways
hsa00061	Fatty acid biosynthesis
hsa04910	Insulin signaling pathway
hsa04920	Adipocytokine signaling pathway
hsa04060	Cytokine-cytokine receptor interaction
hsa04630	Jak-STAT signaling pathway
hsa04080	Neuroactive ligand-receptor interaction
hsa00360	Phenylalanine metabolism
hsa00350	Tyrosine metabolism
hsa00760	Nicotinate and nicotinamide metabolism
hsa04920	Adipocytokine signaling pathway
hsa04610	Complement and coagulation cascades
hsa04920	Adipocytokine signaling pathway
hsa03320	PPAR signaling pathway
hsa04610	Complement and coagulation cascades
hsa04115	p53 signaling pathway
hsa04610	Complement and coagulation cascades
hsa04512	ECM receptor interaction
hsa04510	Focal adhesion

## Data Availability

The data used to support the findings of this study are available from the corresponding author upon request.
